# Dengue Disease Severity in Pediatric Patients With Different Blood Groups: A Study at a Tertiary Care Hospital in Jharkhand

**DOI:** 10.7759/cureus.77940

**Published:** 2025-01-24

**Authors:** Upendra P Sahu, Nimisha Vatsana, Suman Kumar, Varun Garg, Omar Hasan

**Affiliations:** 1 Department of Pediatrics, Rajendra Institute of Medical Sciences, Ranchi, IND

**Keywords:** abo blood groups, blood group susceptibility, dengue haemorrhagic fever (dhf), dengue shock syndrome (dss), dengue thrombocytopenia, disease severity predictors, hematological parameters in dengue, igm and ns1 antigen, prognostic markers in severe dengue, rh factor

## Abstract

Background: Dengue fever (DF) is a serious public health concern, especially in tropical regions like India. Cases cluster during the monsoon season due to the proliferation of Aedes mosquitoes, significantly burdening public health and the economy. In addition to prevention and control, identifying risk factors that provide early indications of progression to severe forms of the disease can help in prognostication and improve case management. This study aims to explore the association between ABO blood groups and the severity of dengue disease in pediatric patients admitted to a tertiary care hospital in Jharkhand.

Methodology: This hospital-based observational cross-sectional study enrolled 88 patients, aged 1 to 18 years, with laboratory-confirmed dengue. Clinical features, hematological parameters, and outcomes, including DF, dengue hemorrhagic fever (DHF), and dengue shock syndrome (DSS), were analyzed across different blood groups.

Results: A higher prevalence of dengue was noted in patients with blood group O (33, 37.5%) compared to other blood groups. While blood group O was associated with higher rates of severe outcomes such as DHF in 12 (46.1%) patients and DSS in 9 (56.2%) patients, statistical analysis did not establish a significant association between ABO blood groups and dengue severity (*P* = 0.155). Common presenting symptoms included abdominal pain in 61 (69.3%), body ache in 57 (64.8%), and fever in 50 (56.8%) patients, while complications like vascular leakage and thrombocytopenia varied across blood groups.

Conclusions: While no definitive link between blood group and disease progression was found, the result of higher prevalence of severe disease in blood group O can help us to stay vigilant. Identifying predictive markers for severe dengue can enhance handling of these patients and improve morbidity and mortality in pediatric populations. This study underscores the need for further research into the genetic and immunological factors that influence dengue severity.

## Introduction

Dengue virus is a global health challenge, with a significant impact on Southeast Asia, the Western Pacific, and the Americas [1]. In India, dengue is a major public health concern, contributing to substantial morbidity and mortality [2]. The disease imposes an economic burden through loss of productivity and affects the quality of life of affected individuals. Effective prevention, early detection, and timely management are critical to minimizing the progression of dengue into severe complications such as dengue hemorrhagic fever (DHF) and dengue shock syndrome (DSS) [3]. 

Dengue fever (DF) is defined as an acute febrile illness lasting more than 2 days but less than 10 days, with two or more manifestations, including retroorbital pain, severe headache, severe muscular pain, severe backache, myalgias/arthralgias, joint pain, and a platelet count of less than 150,000. DHF is defined by the presence of all of the following: (1) platelet count ≤100,000/μL; (2) acute febrile illness of >2 days but <10 days; (3) at least one of the following features as evidence of hemorrhage phenomenon: (a) purpura, ecchymoses, or petechiae; (b) bleeding from the gastrointestinal tract, injection sites, mucosa, or other sites; (c) a positive tourniquet test; (d) melena or hematemesis; (4) at least one of the following features as evidence of plasma leakage: (a) pleural effusion or ascites, or evidence of hypoalbuminemia (plasma leakage signs); (b) an increase in hematocrit 20% from previous hematocrit (hemoconcentration). DSS includes DHF plus evidence of circulatory failure: pulse pressure ≤20 mmHg with a feeble and weak pulse, or manifested by cold and clammy skin, hypotension, or irritability.

The potential relationship between blood groups and susceptibility to diseases was first suggested by Kaipainen et al. in 1960 [4]. The ABO blood grouping system, a key component of innate immunity, influences an individual's susceptibility and resistance to various infections [5]. Natural IgM antibodies, which target glycosylated antigens missing from specific blood groups, may cross-react with glycosylated viral proteins in dengue, potentially exacerbating disease severity [6]. This unique interaction has garnered research interest, as studies have demonstrated associations between ABO blood groups and diseases such as cardiovascular conditions, cancers, malaria, and cholera. In the context of dengue, some studies have reported a higher prevalence in specific blood groups, while others suggest links between blood group type and disease severity [1].

Dengue, commonly referred to as "breakbone fever," is a mosquito-borne disease transmitted by Aedes mosquitoes. Four strains of the virus - DENV 1, DENV 2, DENV 3, and DENV 4 - are responsible for the infection [7]. India's tropical climate, particularly during the monsoon season, creates favorable conditions for the proliferation of the Aedes vector, leading to a significant increase in cases in both urban and rural regions [8]. The clinical spectrum of dengue ranges from asymptomatic infections to severe manifestations like DHF and DSS. Following an incubation period of two to seven days, the disease typically presents with sudden fever, headache, muscle pain, joint pain, and a rash [9]. Early detection methods, such as NS1 antigen testing, are vital for timely intervention, as IgM antibodies only become detectable after six days of fever onset [10].

Several factors influence the severity of dengue, including age, genetics (such as human leukocyte antigen [HLA] and ABO blood groups), viral strain, nutritional status, and secondary infections [11]. Newer vaccines, such as Dengvaxia and Qdenga, have proven effective against dengue infection; however, their availability in our state is limited. Identifying early markers of severity remains a priority in research to improve disease outcomes. This study aims to investigate the association between ABO blood groups and dengue severity, with the goal of enhancing our understanding of disease progression and contributing to improved clinical management strategies [12]. No study has been conducted in Jharkhand to correlate clinical and hematological parameters with disease severity. Therefore, this study aims to establish a relationship for future reference.

## Materials and methods

Study design

This was a hospital-based observational cross-sectional study conducted to assess the association between ABO blood groups and the severity of dengue disease in pediatric patients.

Study duration and population

The study was carried out over a period of 12 months at the Department of Pediatrics, Rajendra Institute of Medical Sciences (RIMS), Ranchi. The study involved pediatric patients aged 1-18 years who were admitted with clinical and laboratory-confirmed DF. The study was done from April 2023 to April 2024 after receiving formal approval from the Institutional Ethics Committee, under all the ethical standards.

Study site

The study was conducted in the pediatric ward of the Department of Pediatrics at RIMS, Ranchi.

Inclusion criteria

The study included patients aged 1-18 years who were diagnosed with DF based on clinical features and laboratory confirmation. Only those patients who were willing to participate in the study were included.

Exclusion criteria

Patients younger than one year were excluded from the study, as were those with prolonged fever cases unrelated to dengue. Additionally, patients with generalized edema due to renal, cardiac, or liver pathology, or with petechial spots resulting from bone marrow failure, malignancy, or other systemic disorders, were excluded. Patients who were unwilling to participate were also excluded from the study.

Sample size

A total of 88 patients were included in the study, which was based on previous hospital admission data for dengue cases over the past three years.

Sample collection and testing

Blood samples were collected from all patients upon admission using standard aseptic techniques. These samples were analyzed for a range of diagnostic tests, including complete blood count (CBC) using both automated and manual methods. Blood grouping for ABO and Rh factors was performed using anti-A, anti-B, and anti-D reagents. Liver function tests (LFTs), renal function tests (RFTs), and NS1 antigen detection were conducted to aid in early diagnosis of dengue. Additionally, IgM antibodies were tested after the acute phase for confirmation of DF. Ultrasonography (USG) of the abdomen and chest was performed to identify third-space fluid accumulation, while Hess’s capillary fragility test, also known as the tourniquet test, was used to assess capillary leakage.

Data collection

Data were collected using a pre-tested, semi-structured questionnaire, which included questions on sociodemographic details, clinical features, symptom history, and treatment history. Laboratory and radiological findings were also recorded to assist in understanding the disease progression and severity in the patients.

Statistical analysis

The collected data were entered into MS Excel and analyzed using SPSS software (version 20.0, IBM Corp., Armonk, NY). Continuous variables were expressed as mean ± standard deviation (SD), while ordinal data were presented as median ± interquartile range (IQR). Appropriate parametric and nonparametric statistical tests were applied to determine any associations between ABO blood groups and the severity of dengue.

## Results

Age and DF

The study included 88 pediatric patients, with a mean age of 8.44 ± 3.71 years (range: 1-18 years). This indicates that school-aged children are more affected by the dengue virus, likely due to higher exposure from their play activities and increased mobility, which expose them to more mosquito bites.

Gender and DF

In this study, there were 45 (51.13%) male and 43 (48.86%) female patients. There is a near-equal distribution of DF among both the genders. 

Clinical features and DF

**Figure 1:**
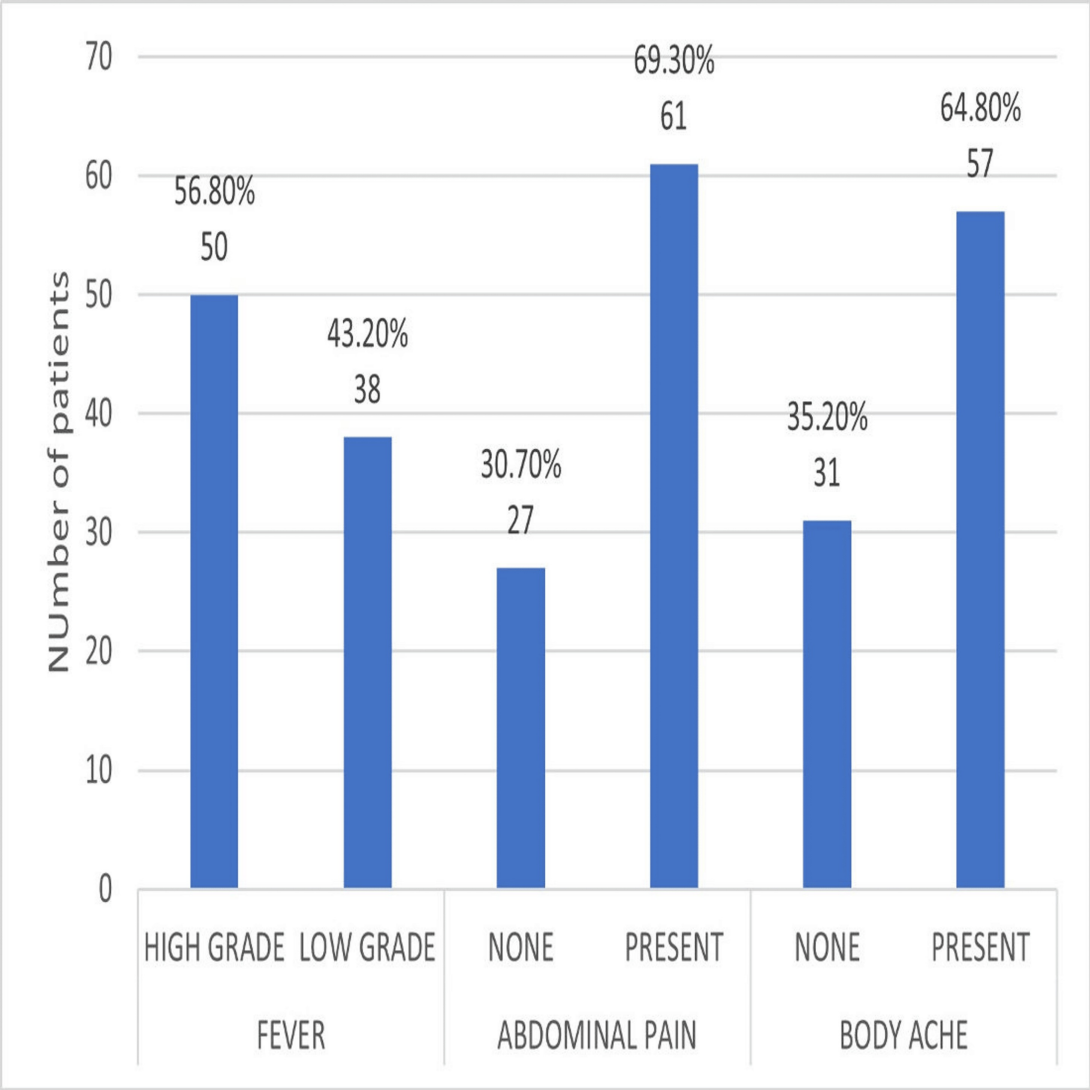
Analysis of clinical features.

The most common presenting features are abdominal pain (61, 69.3%) and body ache (57, 64.8%), as depicted in Figure 1. Fever is the next common symptom. It represents the classical clinical features of DF. The higher incidence of abdominal pain and body ache can be attributed to the study being conducted in a tertiary care hospital.

Bleeding manifestation and DF 

**Figure 2:**
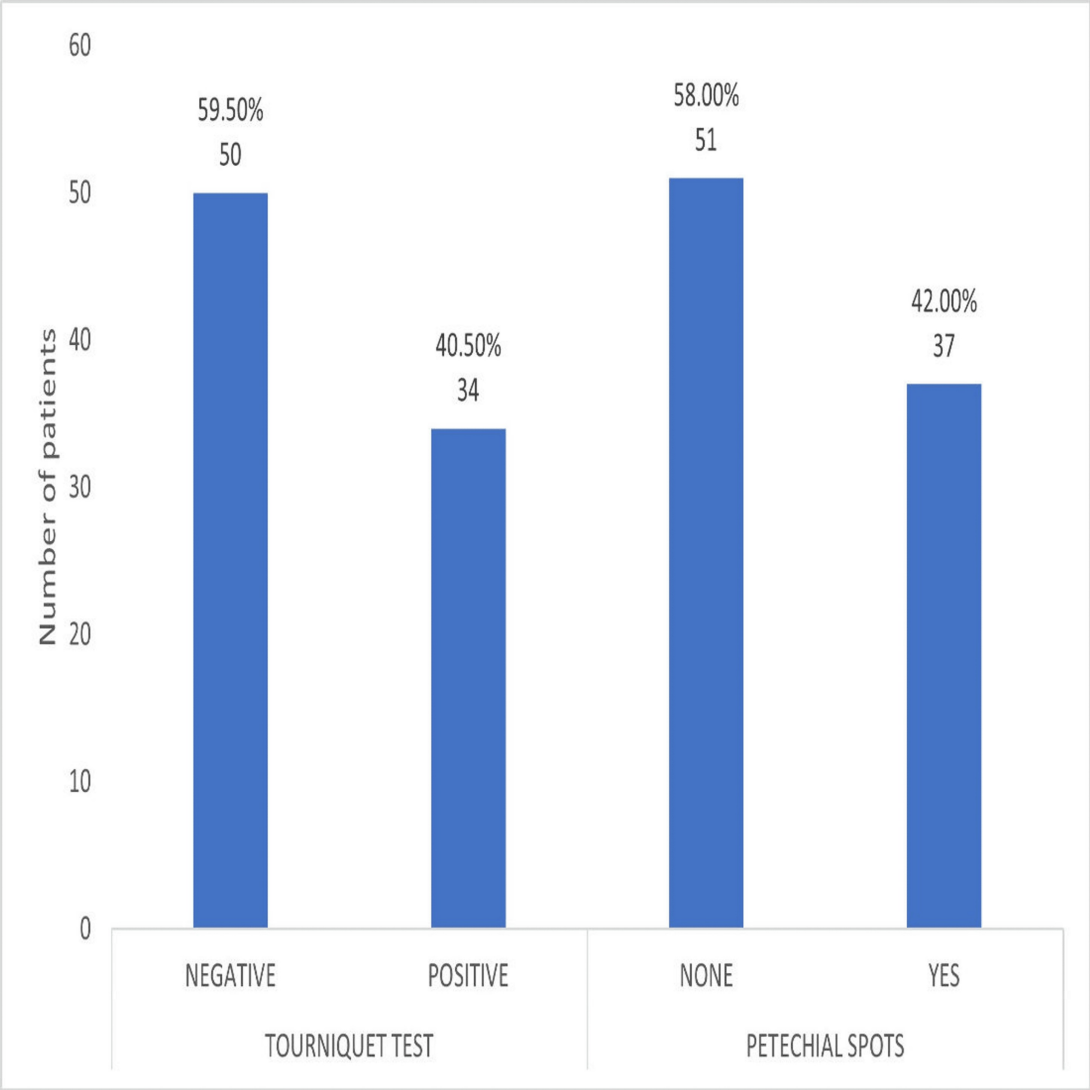
Comparison of bleeding manifestations.

Only 37 (42%) patients presented with complaints of petechial spots, while 34 (40.5%) patients had a positive tourniquet test at the time of admission or during their hospital stay, as depicted in Figure 2. Although there was lesser incidence of bleeding among the patients, it is an early marker to asses the severity of the disease.

Vascular leakage and DF

**Figure 3:**
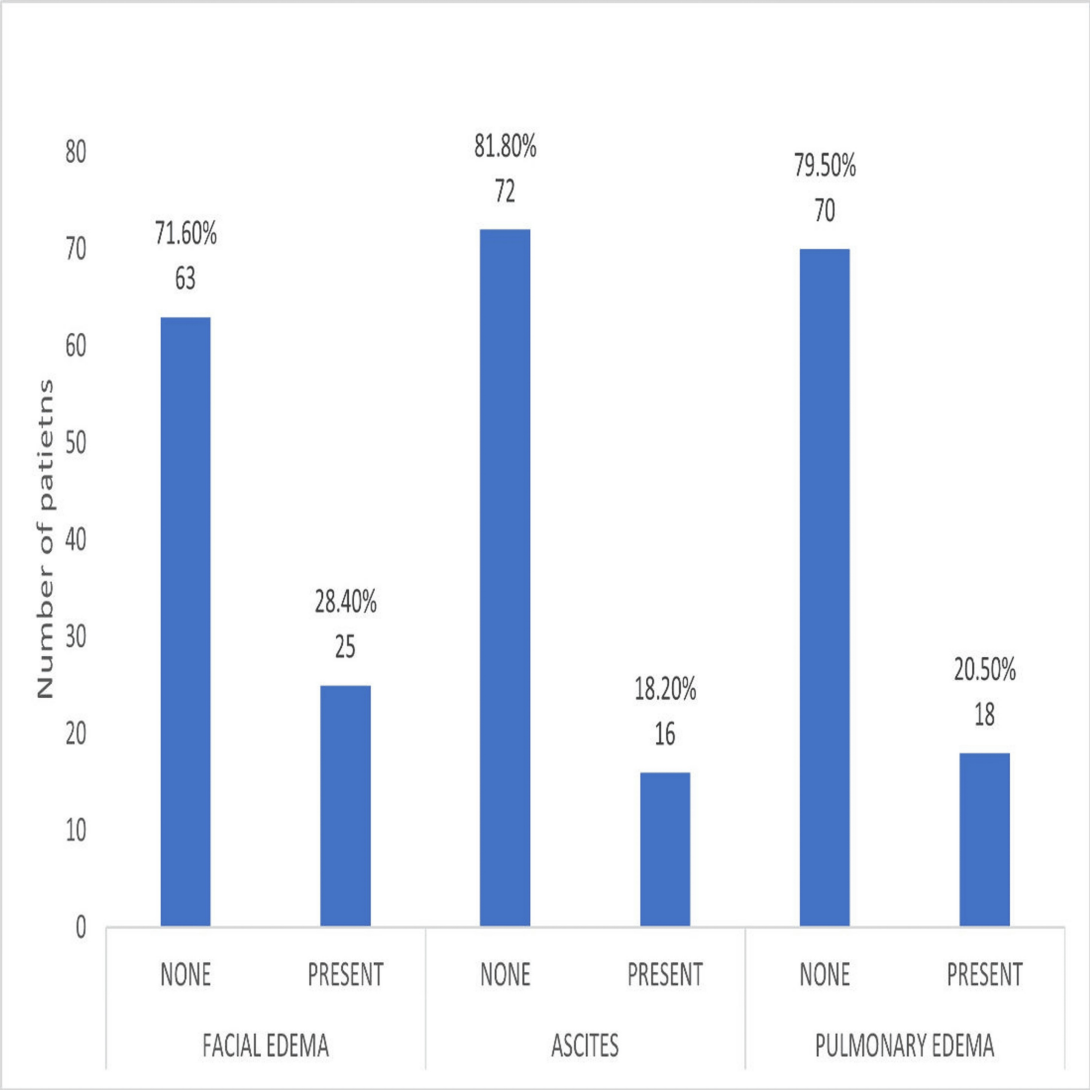
Comparison of vascular leakage phenomenon.

Vascular leakage was observed in the form of facial edema in 25 (28.4%) patients, ascites in 16 (18.2%) patients, and pulmonary edema in 18 (20.5%) patients, as shown in Figure 3.

DSS occurred in only 16 (18.2%) patients while DHF was seen in 26 (29.5%) patients. DF had the maximum incidence and was seen in 46 (52.3%) patients. DF is found to be the most common form of dengue infection suggesting that most of the diseases are mild forms and DSS, the most severe one affects only the more suspectable population. 

Blood group and dengue disease

**Figure 4:**
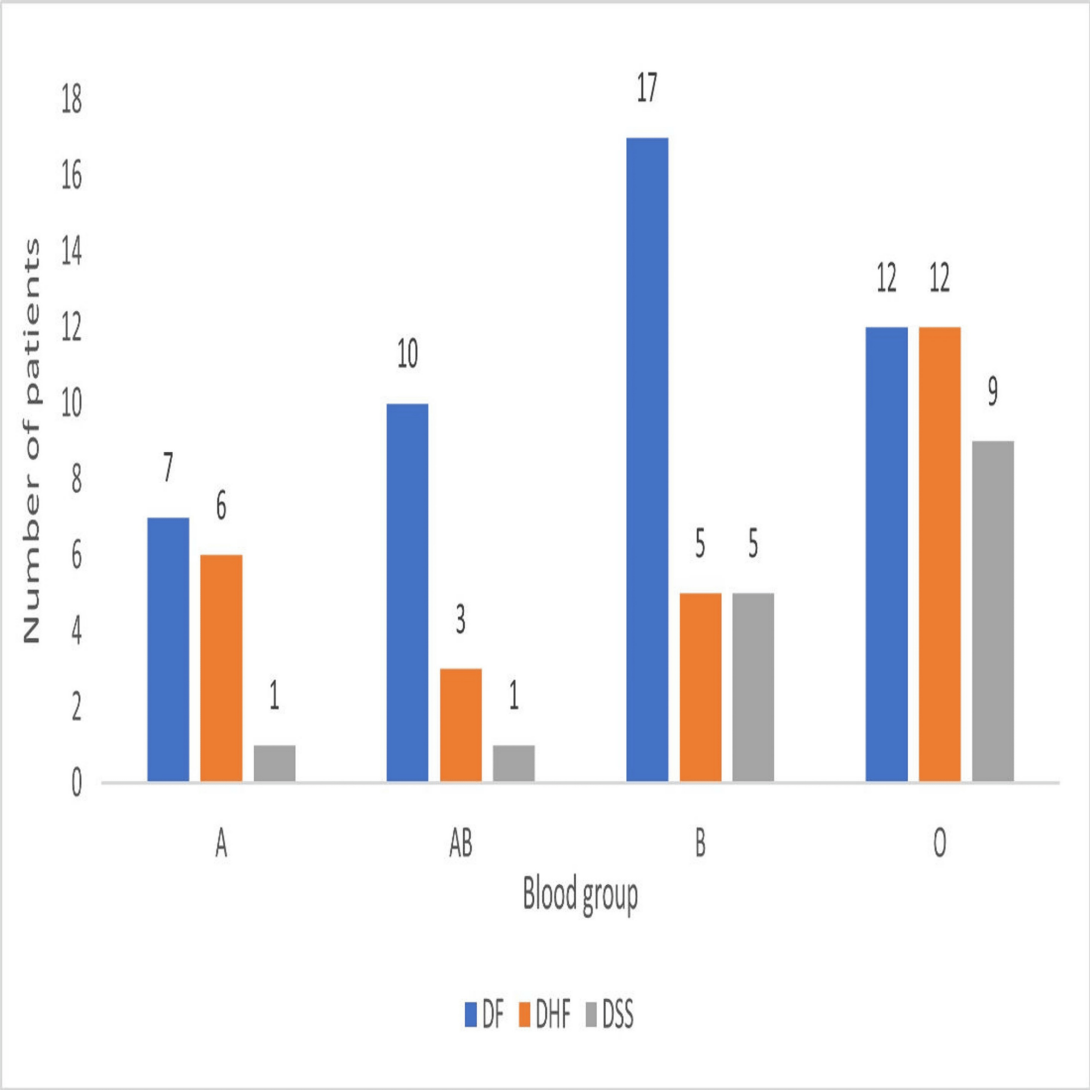
Comparison of outcomes with various blood groups. DF, dengue fever; DHF, dengue hemorrhagic fever; DSS, dengue shock syndrome

Patients with blood group O were the most affected by the dengue virus (33, 37.5%), while blood groups A and AB were the least affected, with 14 (15.9%) patients each (Figure 4).

DF occurred most in blood group B (17, 36.9%), while DHF occurred most in blood group O (12, 46.1%). DSS was most seen in blood group O (9, 56.2%) (Table 1).

**Table 1:** Outcomes in various blood groups. DF, dengue fever; DHF, dengue hemorrhagic fever; DSS, dengue shock syndrome

Blood group	DF	DHF	DSS	Total
A	7	6	1	14
AB	10	3	1	14
B	17	5	5	27
O	12	12	9	33
Total	46	26	16	88

On further analysis for the strength of association of DF with different blood groups, no significant association was found between the two (chi-square = 9.345, *P*-value = 0.155).

Descriptive statistics of various hematological parameters 

In this study, the mean RBC count was 4.22 ± 0.97 lakhs per microliter. The total leukocyte count had a mean of 6,582.9 ± 4,052.6 cells per microliter. The mean hemoglobin level (g/dL) was 10.37 ± 1.61. The mean AST was 192.66 ± 169.49. The mean ALT was 75.89 ± 70.58. In this study, the mean albumin level was 4.008 ± 0.50. Mean urea levels were 41.71 ± 24.76 mg/dL, and mean creatinine levels were 0.9 ± 0.36 mg/dL (Table 2).

**Table 2:** Descriptive statistics of various hematological parameters. AST, aspartate aminotransferase; ALT, alanine aminotransferase

Parameters	Mean	Standard deviation	Minimum	Maximum
Platelet (10⁵ cells/microliter)	1.751	1.008	0.230	4.400
Red blood cell (lakhs/microlitre)	4.221	0.972	1.13	6.4
Total lymphocyte count (cells/microliter)	6,582.955	4,052.616	2,000	33,270
Haemoglobin (g/dL)	10.375	1.614	6.4	13.7
Haematocrit %	36.2	5.464	24.6	46.8
Total bilirubin (mg/dL)	0.762	0.35	0.34	2.2
Direct bilirubin (mg/dL)	0.285	0.143	0.1	0.8
AST (U/L)	192.666	169.492	34.5	1331.8
ALT (U/L)	75.89	70.586	13.6	378
Albumin (mg/dL)	4.008	0.505	3.2	4.96
Urea (mg/dL)	41.715	24.763	11	92.5
Creatinine (mg/dL)	0.903	0.363	0.22	1.5

Description of various parameters with respect to blood group is given in Table 3. Upon analyzing the positive clinical features in relation to blood group, it was found that patients with blood group O had a higher number of cases with high-grade fever (22, 44%). Abdominal pain was present in 22 (61%) patients, body ache in 19 (33.33%) patients, hepatomegaly in 7 (41.1%) patients, a positive tourniquet test in 14 (41.1%) patients, petechial spots in 15 (40.5%) patients, facial edema in 13 (52%) patients, and pulmonary edema in 7 (38.8%) patients. However, none of these clinical features were clinically significant.

**Table 3:** Description of various parameters with respect to blood groups. df, degrees of freedom; F, female; M, male

Parameters		Blood group				Total	*χ*^2^ value	df	P
		A	AB	B	O				
Sex	F	7	8	13	15	43	0.55	3	0.908
	M	7	6	14	18	45			
Fever	High grade	7	5	16	22	50	4.177	3	0.243
	Low grade	7	9	11	11	38			
Abdominal pain	None	4	4	8	11	27	0.182	3	0.98
	Present	10	10	19	22	61			
Body ache	None	4	3	10	14	31	2.228	3	0.526
	Present	10	11	17	19	57			
NS1 antigen	Negative	5	9	7	10	31	6.557	3	0.087
	Positive	9	5	20	23	57			
IgM antibody	Negative	5	4	10	14	33	0.839	3	0.84
	Positive	9	10	17	19	55			
Hepatomegaly (enlarged >2 times)	No	13	12	20	26	71	2.391	3	0.495
	Yes	1	2	7	7	17			
Tourniquet test	Negative	9	6	16	19	50	2.683	3	0.443
	Positive	4	8	8	14	34			
Petechial spots	None	10	7	16	18	51	1.583	3	0.663
	Yes	4	7	11	15	37			
Facial edema	None	9	12	22	20	63	4.997	3	0.172
	Present	5	2	5	13	25			
Ascites	None	12	12	23	25	72	1.306	3	0.728
	Present	2	2	4	8	16			
Pulmonary edema	None	11	11	22	26	70	0.09	3	0.993
	Present	3	3	5	7	18			
Hypotension	Mild	4	2	6	5	17	8.563	9	0.479
	None	8	12	18	19	57			
	Moderate	2	0	2	7	11			
	Severe	0	0	1	2	3			
Outcome	DF	7	10	17	12	46	9.345	6	0.155
	DHF	6	3	5	12	26			
	DSS	1	1	5	9	16			

## Discussion

DF continues to pose a significant public health challenge, particularly in tropical regions like India, where the monsoon season exacerbates the proliferation of Aedes mosquitoes, leading to an increase in disease burden [13]. Understanding genetic factors, including the potential association between ABO blood groups and dengue severity, is crucial for identifying individuals at higher risk of developing severe outcomes, such as DHF and DSS.

In our study, blood group O, dengue was found to be the most prevalent (33, 37.5%) among the pediatric patients, and it was associated with higher rates of DHF (12, 46.1%) and DSS (9, 56.2%). These findings are consistent with previous studies, such as one conducted in India in 2010, which reported a higher prevalence of dengue among individuals with blood group O [14]. However, conflicting results have also been observed in other studies. For instance, a 2018 study suggested a lower likelihood of DF among individuals with blood group O [15].

The potential role of ABO blood groups in influencing the severity of dengue may stem from the glycosylation of viral proteins. Natural IgM antibodies present in individuals with blood group O may cross-react with glycosylated viral proteins, potentially increasing disease severity by triggering immune responses [16]. While this theoretical basis supports the observations of more severe cases in individuals with blood group O, our study did not find a statistically significant association between ABO blood groups and dengue severity (*P* = 0.155). This finding aligns with studies such as that of Khode et al., who also reported no significant link between ABO blood group and disease severity [17]. Various studies are ongoing in the field of microbiology to find other causes of this association. 

Regarding clinical features, abdominal pain among 61 (69.3%) patients and body ache in 57 (64.8%) patients were the most common symptoms observed in our cohort, consistent with the known presentation of DF. Complications like vascular leakage, as evidenced by facial edema in 25 (28.4%), pulmonary edema in 18 (20.5%), and ascites in 16 (18.2%) patients, reflects the critical need for early recognition of warning signs to prevent progression to severe forms of the disease. Additionally, thrombocytopenia, which is a hallmark of severe dengue, was most pronounced in patients with blood group O, highlighting its relevance as a key indicator of disease progression. This could be advantageous to mankind when used in conjunction with AI to predict the course of the disease and alert us for early intervention.

Despite these findings, our study has limitations that should be considered. The single-center design and relatively small sample size may limit the generalizability of our results. Furthermore, other genetic and environmental factors influencing disease severity, such as HLA typing and the impact of secondary infections, were not explored in depth. Future multicenter studies with larger cohorts and more comprehensive genetic profiling are needed to validate and expand upon these findings. Conducting the study exclusively in a tertiary care hospital could introduce bias, as patients in such facilities may differ from those in primary or community care settings. Hematological parameters may vary depending on the exact day of the febrile illness on which samples were taken, which can affect the study findings. Standardized timing in future studies would help ensure more consistent data. The presence of other hemorrhagic viruses could not be thoroughly investigated, which may have influenced clinical progression and laboratory parameters. The inability to measure dengue-specific IgG antibodies limited the assessment of secondary infections and their effect on disease severity. Severe dengue manifestations often occur during the defervescence period. Analyzing severity markers specifically around the time of defervescence, rather than only on the day of admission, could yield more accurate insights. Differences in the processing of samples, such as using plasma versus serum, can affect levels of severity markers. Standardization of sample collection and processing protocols is recommended to reduce variability and improve comparability.

In conclusion, while blood group O showed a higher prevalence and was associated with greater severity of dengue in our study, no significant association was established between ABO blood groups and dengue severity. Similar results were also found in adults. Continued research into genetic, immunological, and clinical predictors of severe dengue is essential to improve patient outcomes and tailor effective public health interventions.

## Conclusions

This study aimed to explore the association between ABO blood groups and the severity of dengue disease in pediatric patients. While blood group O was found to have the highest prevalence (33, 37.5%) and was associated with more severe outcomes such as DHF and DSS, no statistically significant relationship was observed between ABO blood groups and disease severity (*P* = 0.155).

Common clinical presentations, including abdominal pain, fever, and body ache, were noted across all blood groups, along with complications like vascular leakage and thrombocytopenia. However, thrombocytopenia was particularly pronounced in patients with blood group O, highlighting the need for vigilant monitoring and clinical attention in this group.

Although no definitive association between ABO blood groups and dengue severity was established in this study, the findings underscore the importance of further large-scale, multicenter research. Such studies could help identify genetic and immunological predictors of disease progression, potentially leading to improved clinical management and more targeted interventions for severe dengue cases in pediatric populations.
